# Overall Survival With Pyrotinib Plus Capecitabine Versus Lapatinib Plus Capecitabine in HER2‐Positive Metastatic Breast Cancer: A Single‐Center Pooled Analysis

**DOI:** 10.1111/1759-7714.70362

**Published:** 2026-08-06

**Authors:** Jialin Lin, Qiao Li, Pin Zhang, Ying Fan, Ruigang Cai, Yang Luo, Bo Lan, Shanshan Chen, Jiani Wang, Hongnan Mo, Fei Ma, Jiayu Wang, Binghe Xu

**Affiliations:** ^1^ Department of Medical Oncology National Cancer Center, Cancer Hospital, Chinese Academy of Medical Sciences and Peking Union Medical College Beijing China; ^2^ Beijing Key Laboratory of Innovative Drug R&D and Translational Research for Breast Cancer Beijing China

**Keywords:** HER2‐positive, metastatic breast cancer, overall survival, pyrotinib, TKI

## Abstract

**Background:**

Phase II and III trials have demonstrated progression‐free survival (PFS) benefits of pyrotinib plus capecitabine over lapatinib plus capecitabine in HER2‐positive metastatic breast cancer (MBC). However, long‐term overall survival (OS) data from a single‐center cohort remain limited. This pooled analysis compared OS between the two regimens and explored outcomes across prespecified subgroups.

**Methods:**

We included patients with HER2‐positive MBC from our center enrolled in a Phase Ic study, a Phase II study, or the Phase III PHOEBE trial. The primary endpoint was OS. OS was analyzed using Kaplan–Meier estimates, log‐rank tests, and a multivariable Cox model adjusted for ECOG performance status, pathological grade, prior anti‐HER2 therapy, trastuzumab exposure duration, trastuzumab resistance, and prior chemotherapy lines. The proportional hazards assumption was assessed using Schoenfeld residuals. Exploratory subgroup analyses used prespecified categories.

**Results:**

At data cutoff, 82 patients were included; 53 received pyrotinib plus capecitabine and 29 received lapatinib plus capecitabine. Baseline characteristics were comparable between groups. Median OS was 74.61 months (95% CI 41.10–not reached) with pyrotinib plus capecitabine and 30.98 months (26.12–50.76) with lapatinib plus capecitabine (log‐rank *p* = 0.0053). Cox models suggested a reduced risk of death with pyrotinib plus capecitabine. Subgroup analyses were exploratory and should be interpreted cautiously because several subgroup estimates were imprecise.

**Conclusion:**

In this single‐center pooled analysis, pyrotinib plus capecitabine was associated with significantly longer OS than lapatinib plus capecitabine in patients with HER2‐positive MBC. These exploratory results are consistent with prior Phase II/III trials and provide evidence supporting the OS benefit of pyrotinib.

## Graphical abstract

In this single‐center pooled analysis of HER2‐positive metastatic breast cancer, pyrotinib plus capecitabine was associated with significantly longer overall survival than lapatinib plus capecitabine. This survival benefit remained significant in a sensitivity analysis restricted to the randomized Phase II and III trials and after multivariable adjustment for clinically relevant baseline factors.

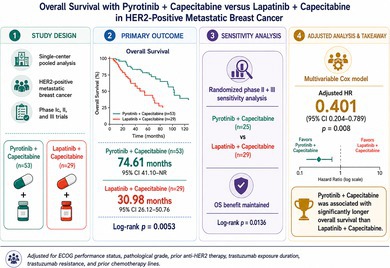

## 1 Introduction

HER2‐positive metastatic breast cancer (MBC) exhibits aggressive biological behavior and is associated with a relatively poor prognosis [1, 2, 3]. The introduction of various HER2‐targeted agents, including trastuzumab, pertuzumab, trastuzumab emtansine (T‐DM1), trastuzumab deruxtecan (T‐DXd), and small‐molecule tyrosine kinase inhibitors (TKIs), has substantially extended median survival compared with the era prior to HER2‐directed therapy [4, 5]. Nevertheless, long‐term overall survival (OS) outcomes for oral TKI‐based regimens, as well as their treatment effects across distinct clinical subgroups, remain incompletely characterized [4, 6].

Lapatinib combined with capecitabine is among the first oral HER2‐directed TKI regimens adopted in clinical practice and has demonstrated activity in several trials. However, both progression‐free survival (PFS) and long‐term OS with this regimen remain suboptimal and warrant further improvement [7, 8, 9]. Pyrotinib, an oral irreversible pan‐ErbB TKI targeting EGFR, HER2, and HER4, was designed to provide more sustained blockade of the HER family compared with reversible TKIs such as lapatinib [6, 10]. In a Phase I trial, pyrotinib plus capecitabine showed acceptable tolerability and preliminary evidence of a high objective response rate (ORR). Subsequent multicenter, randomized Phase II and III PHOEBE trials further established that pyrotinib plus capecitabine significantly prolongs PFS versus lapatinib plus capecitabine in patients with HER2‐positive MBC [6, 10, 11, 12].

At the time of initial reporting, OS follow‐up in these studies remained immature, and long‐term OS outcomes together with patterns of benefit across different clinical subgroups, especially within single‐center cohorts, had not yet been fully characterized [13]. We therefore conducted a pooled analysis of patients enrolled at our center across the Phase Ic, II, and III trials comparing pyrotinib plus capecitabine versus lapatinib plus capecitabine [6, 10, 11]. With OS as the primary endpoint, this analysis aimed to evaluate the long‐term survival difference between the two regimens in our institutional cohort and to explore potential treatment benefit within key clinically subgroups.

## 2 Methods

### 2.1 Study Design and Population

We conducted a single‐center pooled analysis of patients enrolled at our institution across three clinical trials of pyrotinib. These encompassed: (1) a Phase Ic, single‐arm, dose‐escalation, and expansion study evaluating pyrotinib plus capecitabine [10]; (2) a multicenter, randomized, open‐label Phase II trial comparing pyrotinib plus capecitabine to lapatinib plus capecitabine in patients with prior exposure to taxanes, anthracyclines, and/or trastuzumab [11]; and (3) the multicenter, open‐label, randomized Phase III PHOEBE trial, which compared the same two regimens in patients with HER2‐positive MBC previously treated with trastuzumab and taxanes [6]. The study designs and primary outcomes have been reported elsewhere. All patients enrolled and treated at our center in these trials were included in the present analysis.

Patients' eligibility in the original trials was defined by the following key criteria: histologically or cytologically confirmed HER2‐positive metastatic or recurrent breast cancer (IHC 3+ or 2+ with positive in situ hybridization), prior therapy with at least one anthracycline and/or taxane containing chemotherapy regimen, an Eastern Cooperative Oncology Group (ECOG) performance status of 0 or 1, the presence of at least one measurable lesion, and adequate organ function. Major exclusion criteria included intolerance to capecitabine, prior exposure to small‐molecule HER2 TKIs, a history of significant cardiovascular disease, active central nervous system metastases, and any concurrent condition that, in the investigator's judgment, would compromise patient safety or protocol compliance. Detailed inclusion and exclusion criteria for each trial have been reported previously [6, 10, 11].

All three parent studies were approved by the institutional ethics committee and were conducted in accordance with the Declaration of Helsinki and Good Clinical Practice guidelines. All participants provided written informed consent before enrollment. The pooled analysis, which entailed additional follow‐up and secondary evaluation of data from patients treated at our center, was also reviewed and approved by the same ethics committee.

### 2.2 Treatment and Procedures

In all three source trials, patients assigned to the pyrotinib plus capecitabine regimen received oral pyrotinib once daily on a continuous schedule together with oral capecitabine twice daily on days 1–14 of each 21‐day cycle. In the Phase Ic study, a dose‐escalation and expansion strategy was used to determine the recommended dose of pyrotinib in combination with capecitabine; the subsequent Phase II and III trials used the recommended dose regimen. For the purposes of this pooled analysis, the actual pyrotinib dose received by each patient was recorded [6, 10, 11].

In the lapatinib plus capecitabine regimen used in the randomized Phase II and III trials, lapatinib 1250 mg was administered orally once daily on a continuous schedule, together with capecitabine 1000 mg/m^2^ twice daily on Days 1–14 of each 21‐day cycle. In both treatment groups, study therapy was continued until disease progression, unacceptable toxicity, death, withdrawal of consent, or investigator decision [6, 11].

### 2.3 Follow‐Up and Endpoints

The primary endpoint of this pooled analysis was OS, defined as the time from the initiation of study treatment in the original trial to death from any cause. Patients who were alive or lost to follow‐up at the data cutoff were censored at their last known contact date. Secondary analyses focused on OS within clinically relevant subgroups. Tumor response and PFS, which were assessed per RECIST version 1.1 and the respective parent trial protocols, were not re‐analyzed in detail in this report [6, 10, 11].

Patient follow‐up was conducted through a combination of routine outpatient visits, review of inpatient medical records, and structured telephone interview. Information on vital status, disease progression, and subsequent anticancer therapies was collected prospectively. A common data cutoff date (September 17, 2025) was prespecified for this analysis, and all available data from the three trials at our center up to this date were included.

### 2.4 Statistical Analysis

Baseline demographic and clinical characteristics were summarized descriptively for each treatment group and for the overall pooled cohort. OS was estimated using the Kaplan–Meier method, and 95% confidence intervals (CIs) for median OS were calculated using standard methods. Comparisons of OS between treatment groups were performed using two‐sided log‐rank tests. Hazard ratios (HRs) and 95% CIs for OS were estimated using Cox proportional hazards regression models. To address potential imbalance in clinically relevant baseline factors, a multivariable Cox model was constructed including treatment group, ECOG performance status, pathological grade, prior anti‐HER2 therapy for advanced disease, trastuzumab exposure duration in the metastatic setting, trastuzumab resistance, and number of previous lines of chemotherapy for metastatic disease. Adjusted HRs with 95% CIs were reported for all covariates included in this model. The proportional hazards assumption was assessed for all covariates using Schoenfeld residuals.

Prespecified subgroup analyses of OS were exploratory and were displayed as forest plots. Subgroups were defined according to age, body mass index, ECOG performance status, hormone‐receptor status, metastatic status at enrollment, pathological grade, prior anti‐HER2 therapy, trastuzumab resistance, metastatic trastuzumab exposure, interval from surgery to recurrence or metastasis, and previous lines of chemotherapy for metastatic disease. All statistical tests were two‐sided, with *p*‐values less than 0.05 considered statistically significant, and analyses were performed using R software (R Foundation for Statistical Computing, Vienna, Austria).

## 3 Results

### 3.1 Patients

**FIGURE 1:**
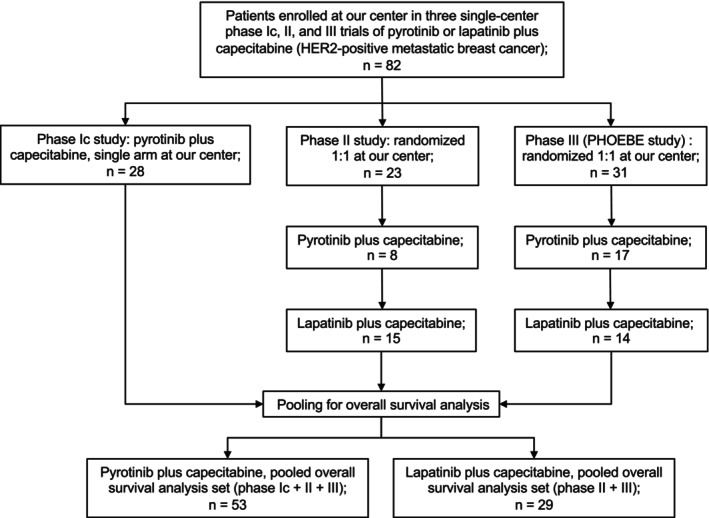
Trial profile of the pooled overall survival analysis. This pooled overall survival analysis included 82 patients enrolled at our center across the Phase Ic, II, and III trials. A total of 53 patients in the pyrotinib plus capecitabine group and 29 patients in the lapatinib plus capecitabine group were included in the pooled overall survival analysis set.

At the data cutoff (September 17, 2025), a total of 82 patients with HER2‐positive MBC from our center were eligible and included in the OS analysis set (Figure 1). These patients were enrolled across three trials of pyrotinib or lapatinib plus capecitabine. Specifically, 28 patients were from the single‐arm Phase Ic study (all receiving pyrotinib plus capecitabine). In the randomized Phase II and III studies, 23 and 31 patients were enrolled at our center, of whom 8 and 17 patients received pyrotinib plus capecitabine, and 15 and 14 received lapatinib plus capecitabine, respectively. Overall, the analysis included 53 patients in the pyrotinib combination group and 29 in the lapatinib combination group. All patients received at least one dose of study treatment and had available survival follow‐up data.

### 3.2 Baseline Characteristics

Baseline demographic and clinical characteristics are summarized in Table 1. The two treatment groups were generally well‐balanced with respect to age, body mass index, ECOG performance status, hormone‐receptor status, pathological grade, and metastatic status at enrolment, which were also broadly comparable to the populations in the parent trials. Although some numerical differences were observed in variables related to prior anti‐HER2 therapy and chemotherapy, including the use and duration of trastuzumab for advanced disease, metastatic trastuzumab exposure, trastuzumab sensitivity, time from surgery to recurrence or metastasis, and the number of prior lines of chemotherapy for metastatic disease, no clear systemic imbalance was evident between the groups.

**TABLE 1:** Baseline demographic and clinical characteristics of patients in the pooled overall survival analysis set.

	Pyrotinib plus capecitabine group (*n* = 53)	Lapatinib plus capecitabine group (*n* = 29)
Age, years	48 (24–68)	52 (25–69)
BMI, kg/m^2^	24.0 (16.9–31.6)	23.6 (19.7–31.6)
ECOG performance status, *n* (%)		
0	36 (67.9%)	9 (31.0%)
1	17 (32.1%)	20 (69.0%)
Hormone receptor status, *n* (%)		
ER and/or PR positive	26 (49.1%)	14 (48.3%)
ER and PR negative	27 (50.9%)	15 (51.7%)
Pathological grade, *n* (%)		
I	1 (1.9%)	0 (0.0%)
II	25 (47.2%)	11 (37.9%)
III	13 (24.5%)	10 (34.5%)
Unknown	14 (26.4%)	8 (27.6%)
Metastatic status at enrollment, *n* (%)		
Nonmetastatic	12 (22.6%)	4 (13.8%)
Metastatic	41 (77.4%)	25 (86.2%)
Prior trastuzumab for advanced disease, *n* (%)		
No	20 (38%)	17 (58.6%)
Yes	33 (62%)	12 (41.4%)
Duration of prior trastuzumab for advanced disease, months	7.4 (0.8–60.5)	12.0 (7.3–18.7)
Metastatic trastuzumab exposure, *n* (%)^a^		
< 1 year	15 (28.3%)	3 (10.3%)
≥ 1 year	5 (9.4%)	6 (20.7%)
No prior metastatic trastuzumab	33 (62.3%)	20 (69.0%)
Resistance to previous trastuzumab, *n* (%)^b^		
No	30 (56.6%)	21 (72.4%)
Yes	23 (43.4%)	8 (27.6%)
Time from surgery to recurrence or metastasis, months^c^	22.8 (2.6–59.1)	18.3 (3.6–146.9)
Surgery to recurrence or metastasis, *n* (%)		
< 1 year	13 (24.5%)	7 (24.1%)
≥ 1 year	31 (58.5%)	17 (58.6%)
De novo Stage IV or missing	9 (17.0%)	5 (17.2%)
Previous lines of chemotherapy for metastatic disease, *n* (%)		
0	23 (43.4%)	12 (41.4%)
1	17 (32.1%)	12 (41.4%)
≥ 2	13 (24.5%)	5 (17.2%)

*Note:* Data are median (IQR) or *n* (%).

^a^ For metastatic trastuzumab exposure, “< 1 year” and “≥ 1 year” were defined among patients who had received trastuzumab for advanced disease; “no prior metastatic trastuzumab” includes patients who received trastuzumab only in the adjuvant setting or had never received trastuzumab.

^b^ Trastuzumab resistance was defined as relapse during or within 6 months after adjuvant trastuzumab, or progression within 3 months of trastuzumab for metastatic disease.

^c^ Evaluated in patients who had undergone surgery; patients with de novo Stage IV disease or missing values are shown separately.

### 3.3 OS

**FIGURE 2:**
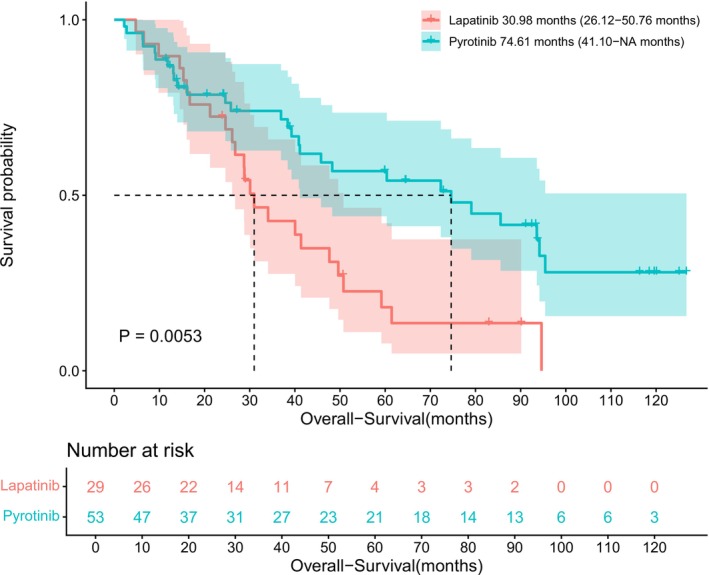
Overall survival in the pooled Phase Ic, II, and III analysis set. Kaplan–Meier curves show overall survival in the pooled analysis set. Median overall survival was 74.61 months in the pyrotinib plus capecitabine group and 30.98 months in the lapatinib plus capecitabine group (*p* = 0.0053).

At data cutoff, the Kaplan–Meier curves demonstrated a sustained separation in OS between the two treatment groups. The median OS was 74.61 months (95% CI 41.10–not reached) in the pyrotinib plus capecitabine group, compared with 30.98 months (26.12–50.76) in the lapatinib plus capecitabine group (log‐rank *p* = 0.0053; Figure 2).

**FIGURE 3:**
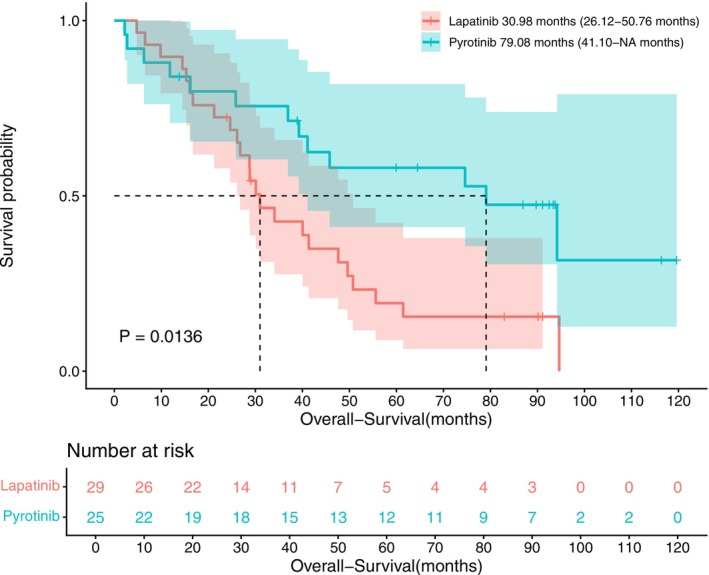
Overall survival in the sensitivity cohort restricted to the randomized Phase II and III trials. Kaplan–Meier curves show overall survival in the sensitivity cohort restricted to the randomized Phase II III trials. Median overall survival was 79.08 months in the pyrotinib plus capecitabine group and 30.98 months in the lapatinib plus capecitabine group (*p* = 0.0136).

In a sensitivity analysis restricted to the randomized Phase II and III trials, pyrotinib plus capecitabine remained associated with significantly longer OS than lapatinib plus capecitabine (log‐rank *p* = 0.0136; Figure 3).

In the multivariable Cox model adjusting for ECOG performance status, pathological grade, prior anti‐HER2 therapy, trastuzumab exposure duration, trastuzumab resistance, and prior chemotherapy lines for metastatic disease, pyrotinib plus capecitabine remained associated with significantly improved OS compared with lapatinib plus capecitabine (adjusted HR 0.401, 95% CI 0.204–0.789; *p* = 0.008; Table 2). Assessment of proportional hazards using Schoenfeld residuals did not indicate a global violation of the proportional hazards assumption (global *p* = 0.153), although a potential deviation from this assumption was observed for trastuzumab resistance (*p* = 0.031; Table S1).

**TABLE 2:** Multivariable Cox proportional hazards analysis for overall survival.

Variable	Adjusted HR	95% CI	*p*
Treatment: pyrotinib vs. lapatinib	0.401	0.204–0.789	0.008
ECOG 1 vs. 0	0.947	0.502–1.787	0.867
Pathological Grade III vs. I–II	1.322	0.657–2.660	0.434
Pathological Grade unknown vs. I–II	1.060	0.512–2.195	0.876
Prior anti‐HER2 therapy: yes vs. no	1.143	0.513–2.547	0.743
Trastuzumab exposure duration, per month	0.939	0.871–1.012	0.100
Trastuzumab resistance: yes vs. no	1.050	0.532–2.074	0.888
Prior chemotherapy lines for metastatic disease: 1 vs. 0	1.079	0.522–2.231	0.837
Prior chemotherapy lines for metastatic disease: ≥ 2 vs. 0	2.213	0.871–5.622	0.095

*Note:* Reference categories: lapatinib plus capecitabine for treatment group, ECOG 0, pathological Grade I–II, no prior anti‐HER2 therapy, no trastuzumab resistance, and 0 prior chemotherapy lines for metastatic disease.

Abbreviations: CI, confidence interval; HR, hazard ratio.

### 3.4 Subgroup Analysis

**FIGURE 4:**
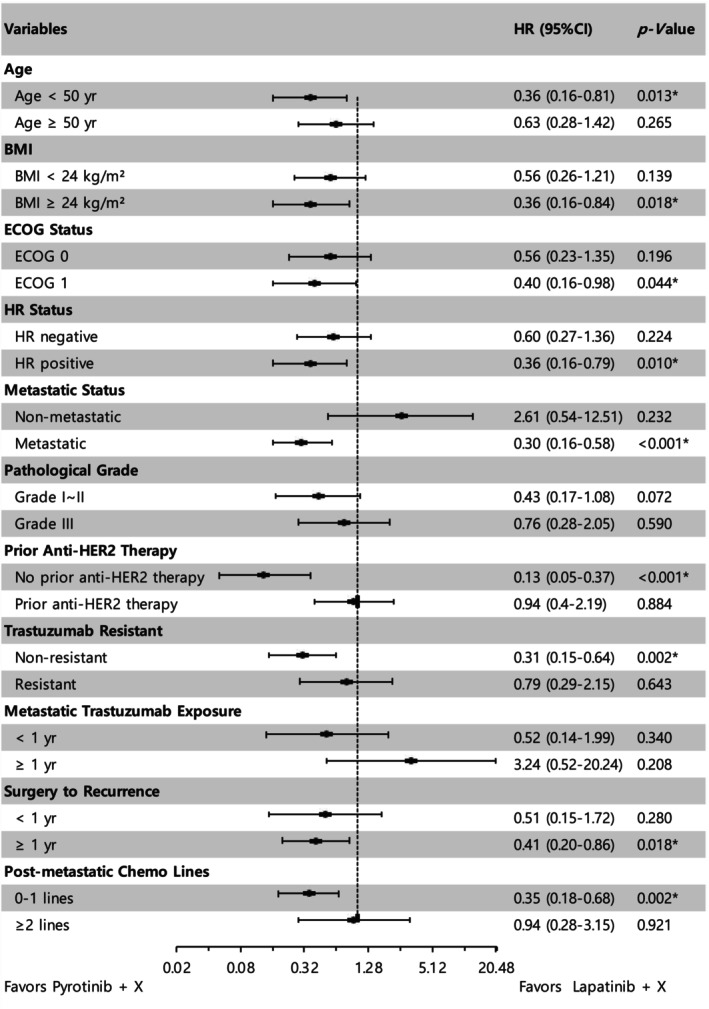
Forest plot of overall survival by clinical subgroup in the pooled Phase Ic, II, and III analysis set. Forest plot showing the effect of pyrotinib plus capecitabine versus lapatinib plus capecitabine on overall survival across prespecified clinical subgroups. Squares indicate hazard ratios, and horizontal lines indicate 95% confidence intervals. HR‐positive disease indicates positivity for estrogen receptor and/or progesterone receptor. Trastuzumab‐resistant disease was defined as relapse during or within 6 months after adjuvant trastuzumab, or progression within 3 months of trastuzumab treatment for metastatic disease. Hazard ratios are from unstratified Cox analyses; values less than 1 favor pyrotinib plus capecitabine over lapatinib plus capecitabine. BMI = body mass index; ECOG = Eastern Cooperative Oncology Group; HR = hazard ratio.

Prespecified subgroup analyses for OS are shown in Figure 4. Point estimates favored pyrotinib plus capecitabine in several subgroups, including patients younger than 50 years, those with a body mass index ≥ 24 kg/m^2^, an ECOG performance status of 1, hormone receptor‐positive disease, metastatic disease at enrollment, no prior anti‐HER2 therapy, trastuzumab‐non‐resistant disease, a surgery‐to‐recurrence or metastasis interval of at least 1 year, and 0–1 previous lines of chemotherapy for metastatic disease. In several other subgroups, including older patients, those with a body mass index < 24 kg/m^2^, ECOG 0, hormone receptor‐negative disease, pathological Grade I–II or III disease, prior anti‐HER2 therapy, trastuzumab‐resistant disease, surgery‐to‐recurrence or metastasis interval < 1 year, metastatic trastuzumab exposure < 1 year, and ≥ 2 previous lines of chemotherapy for metastatic disease, the CIs crossed 1 and the estimates were not statistically precise. Notably, in patients without distant metastases at enrollment and in those with metastatic trastuzumab exposure ≥ 1 year, the HRs were > 1, but the corresponding CIs were very wide; therefore, no reliable subgroup‐specific conclusion can be drawn for these strata. Overall, these subgroup analyses should be considered exploratory.

## 4 Discussion

Our analysis assessed long‐term OS in patients with HER2‐positive MBC treated at our center in three trials comparing pyrotinib versus lapatinib‐based regimens. With extended follow‐up, the pyrotinib plus capecitabine group showed a longer median OS than the lapatinib plus capecitabine regimen. In adjusted multivariable analysis, treatment with pyrotinib plus capecitabine was independently associated with a reduced risk of death. These findings are consistent with the previously reported PFS benefits of pyrotinib and extend the evidence base by providing long‐term, center‐level survival data.

The median OS in this cohort was more than 6 years for patients receiving pyrotinib plus capecitabine, compared with about 2.5 years for those receiving lapatinib plus capecitabine. The direction of this difference corresponds to the PFS separation reported for pyrotinib versus lapatinib‐based regimens in pivotal trials [6, 10, 11, 14, 15, 16]. However, these OS estimates should be interpreted cautiously because differences in prior therapy and access to subsequent HER2‐directed agents may have influenced OS [17]. Because postprogression systemic therapies were not comprehensively captured, the observed OS difference cannot be definitively attributed to the initial TKI regimen alone. Notwithstanding these limitations, our data indicate that pyrotinib‐based therapy may offer a more favorable long‐term disease control profile than lapatinib‐based therapy in this pretreated population. This interpretation is reinforced by meta‐analyses and real‐world evidence demonstrating durable efficacy and encouraging survival outcomes with pyrotinib in advanced, heavily pretreated HER2‐positive MBC, whereas outcomes with lapatinib combinations remain comparatively modest [18, 19, 20, 21]. The long‐term survival trend observed at our center is consistent with the broader published evidence, including the updated Phase III PHILA trial, which reported an OS benefit with pyrotinib plus trastuzumab and docetaxel in untreated HER2‐positive MBC [22]. The subgroup analyses provide additional context for interpreting the overall findings, but they should be regarded as exploratory rather than confirmatory. In our cohort, point estimates favored pyrotinib plus capecitabine in several clinically relevant subgroups, particularly patients with metastatic disease at enrollment, no prior anti‐HER2 therapy, trastuzumab‐non‐resistant disease, a longer interval from surgery to recurrence or metastasis, and 0–1 prior lines of chemotherapy for metastatic disease. These patterns are directionally consistent with the possibility that pyrotinib‐based therapy may be more beneficial when introduced earlier in the metastatic treatment course and in tumors with less evidence of `prior HER2‐directed treatment resistance.

At the same time, the subgroup findings were not uniform. In several strata, including patients with prior anti‐HER2 therapy, trastuzumab‐resistant disease, or ≥ 2 prior lines of chemotherapy for metastatic disease, the HRs were close to 1 and the CIs were wide, indicating substantial uncertainty. More importantly, in patients without distant metastases at enrollment and in those with metastatic trastuzumab exposure ≥ 1 year, the point estimates numerically favored lapatinib plus capecitabine; however, these estimates were highly unstable, with very wide CIs, and should not be overinterpreted. Given the limited sample size and event number within individual strata, these subgroup results do not support firm conclusions regarding differential treatment effect and should be interpreted cautiously.

Any biological interpretation of these subgroup patterns should remain cautious. These clinical features may indicate that the tumor remains predominantly driven by HER2 signaling and that patients were less extensively pretreated with prior systemic therapies. Real‐world evidence from several studies of pyrotinib‐based regimens supports this observation: use in the first‐ or second‐line setting has been associated with longer PFS compared with later‐line use, and some reports also suggest an OS benefit [18, 23, 24, 25]. Similarly, patients who remain sensitive to prior trastuzumab or experience relapse after a longer disease‐free interval tend to achieve better outcomes with subsequent HER2‐targeted therapies, including pyrotinib‐containing combinations [26, 27]. From a biological perspective, these findings align with the rationale that tumors are still primarily dependent on HER2 signaling and not yet equipped with multiple bypass resistance mechanisms are more likely to respond to potent HER2 blockade. This may be especially relevant for irreversible pan‐ErbB inhibitors such as pyrotinib [13, 28, 29, 30, 31]. Further validation of this hypothesis through mechanistic and biomarker‐driven studies is warranted.

By contrast, in subgroups with prior anti‐HER2 therapy, trastuzumab‐resistant disease, or at least two lines of chemotherapy after metastasis, the HRs approached 1 with wide CIs, indicating substantial uncertainty and no clear OS difference. Among patients with prolonged trastuzumab exposure in the metastatic setting, the estimate was particularly unstable, with a very wide CI. This pattern is consistent with clinical experience. Heavily pretreated HER2‐positive MBC, especially after multiple trastuzumab‐based regimens, often develops complex resistance mechanisms. In this context, further intensification with HER2‐targeted TKIs may yield only modest incremental benefit [30, 32, 33]. Real‐world studies of pyrotinib‐based regimens have reported similar trends. Later‐line administration after several prior HER2‐directed therapies is generally associated with shorter PFS and OS, although antitumor activity may still be observed in some lapatinib‐resistant or trastuzumab‐resistant cases [19, 23, 31, 34, 35]. For patients without distant metastases at enrollment, the sample size in our cohort was very small. The resulting estimates were imprecise and the HR exceeded 1; therefore, no reliable conclusion can be drawn for this subgroup. Overall, these subgroup findings should be considered exploratory and hypothesis‐generating. They may help inform future studies evaluating whether pyrotinib‐based therapy is more likely to benefit patients with limited prior anti‐HER2 exposure, maintained sensitivity to trastuzumab‐based therapy, and fewer prior lines of systemic treatment.

Several limitations of this analysis should be acknowledged. First, the study was based on a single‐center pooled cohort with a relatively small sample size, and the number of events in certain subgroups was limited, leading to imprecise estimates of HR. Second, although the contributing trials were generally comparable in terms of treatment regimens and key eligibility criteria, they differed in trial phase, calendar period, and specific operational details. Consequently, variations in prior treatment patterns, postprogression management, and follow‐up schedules across studies may have influenced OS outcomes, and residual confounding cannot be entirely ruled out despite multivariable adjustment. Third, subsequent use of newer HER2‐targeted agents, such as T‐DM1 and T‐DXd, was not comprehensively quantified. Because OS is strongly influenced by postprogression treatment, this limits our ability to distinguish the contribution of initial TKI‐based therapy from that of later‐line treatments to OS, and the observed OS difference cannot be definitively attributed to the initial TKI regimen alone.

## 5 Conclusion

This single‐center pooled analysis of three pyrotinib trials provides further OS data comparing pyrotinib plus capecitabine with lapatinib plus capecitabine in patients with HER2‐positive MBC. Within the limitations of a small, nonrandomized cohort, OS tended to favor the pyrotinib‐based regimen, with indications of benefit across several clinically relevant subgroups. These findings should be regarded as complementary to existing randomized trial evidence rather than conclusive.

## Author Contributions

**Jialin Lin:** funding acquisition, supervision, writing – review and editing, project administration, conceptualization. **Qiao Li:** software. **Shanshan Chen:** data curation. **Fei Ma:** resources, project administration. **Ruigang Cai:** visualization. **Binghe Xu:** funding acquisition, supervision, writing – review and editing, project administration, conceptualization. **Bo Lan:** data curation. **Hongnan Mo:** data curation. **Jiani Wang:** visualization. **Jiayu Wang:** writing – review and editing, methodology. **Ying Fan:** formal analysis. **Yang Luo:** methodology. **Pin Zhang:** investigation.

## Funding

This study was supported with National High Level Hospital Clinical Research Funding (2025‐LYZX‐D‐A02), Noncommunicable Chronic Disease‐National Science and Technology Major Project (2025ZD0552504), and National Natural Science Foundation of China (92459304).

## Conflicts of Interest

The authors declare no conflicts of interest.

## Supporting information


**Table S1:** Assessment of proportional hazards assumption using Schoenfeld residuals.

## Data Availability

The data that support the findings of this study are available from the corresponding author upon reasonable request, subject to privacy and ethical restrictions.
